# Investigating psychological mechanisms linking pain severity to depression symptoms in women cancer survivors at a cancer center with a rural catchment area

**DOI:** 10.1007/s00520-024-08391-9

**Published:** 2024-02-27

**Authors:** Philip I. Chow, Wendy F. Cohn, Patrick H. Finan, David T. Eton, Roger T. Anderson

**Affiliations:** 1 Department of Psychiatry and Neurobehavioral Sciences, Center for Behavioral Health and Technology, University of Virginia School of Medicine, Charlottesville, VA USA; 2 University of Virginia NCI-Designated Comprehensive Cancer Center, Charlottesville, VA USA; 3 Department of Public Health Sciences, University of Virginia School of Medicine, Charlottesville, VA USA; 4 Department of Anesthesiology, University of Virginia School of Medicine, Charlottesville, VA USA; 5 Outcomes Research Branch, Healthcare Delivery Research Program, National Cancer Institute, Bethesda, MD USA

**Keywords:** Depression, Pain, Cancer, Survivors, Women

## Abstract

**Purpose:**

Women cancer survivors, especially those in rural areas, with high levels of depression may be acutely susceptible to pain due to the ways they think, feel, and behave. The current study seeks to elucidate the relationship between symptoms of depression and pain severity in women cancer survivors, by examining the putative mediators involved in this relationship, specifically their self-efficacy for managing their health, how overwhelmed they were from life’s responsibilities, and relational burden.

**Methods:**

Self-report data were collected from 183 cancer survivors of breast, cervical, ovarian, or endometrial/uterine cancer, who were between 6 months and 3 years post-active therapy.

**Results:**

Women cancer survivors with higher (vs. lower) symptoms of depression had more severe pain. Individual mediation analyses revealed that survivors with higher levels of depression felt more overwhelmed by life’s responsibilities and had lower self-efficacy about managing their health, which was associated with greater pain severity. When all mediators were simultaneously entered into the same model, feeling overwhelmed by life’s responsibilities significantly mediated the link between survivors’ symptoms of depression and their pain severity.

**Conclusions:**

The relationship between symptoms of depression and pain severity in women cancer survivors may be attributed in part to their self-efficacy and feeling overwhelmed by life’s responsibilities. Early and frequent assessment of psychosocial factors involved in pain severity for women cancer survivors may be important for managing their pain throughout the phases of cancer survivorship.

## Introduction

Pain is a common and feared symptom experienced by cancer survivors, often caused by cancer or its treatment. Psychological factors have been shown to play a crucial role in modulating pain severity in cancer survivors [1]. In particular, studies have consistently documented a strong positive association between pain and symptoms of depression [2–4]. However, the putative mechanisms underlying this relationship are largely unknown. This paper aims to investigate the pathways between symptoms of depression and pain severity in women cancer survivors who primarily received treatment at a cancer center with a large rural catchment area and completed their active therapy within the past 6 months to 3 years. As a group, women cancer survivors are vulnerable to depression due to the combination of physical effects and changes to body image they experience as a result of cancer treatment, fear of cancer recurrence, and financial-related distress, in addition to other factors that make women twice as likely to develop depression as men [5, 6]. Identifying these pathways may provide valuable insights into developing targeted interventions to alleviate pain severity in this vulnerable population.

### Pain in cancer survivors

Pain is one of the most common long-term symptoms of treatment in cancer survivors [7]. Pain can have a significant impact on cancer survivors, affecting their physical functioning, emotional and psychological well-being, and their ability to adhere to cancer management plans [8, 9]. The biopsychosocial model of pain holds that a variety of factors spanning central and peripheral biology, cultural background, sex, attributions, mood, and personality traits can influence the way pain is expressed by individuals [10, 11]. Studies have found that women experience more pain than men and that pain has a more significant impact on their quality of life [12–14]. In particular, women cancer survivors who live in rural areas may be most impacted by their pain given the paucity of healthcare providers in these regions. Thus, efforts to better predict and treat pain among women cancer survivors must look beyond the proximal cause of initial pain complaints (e.g., chemotherapy) to understand what makes certain women more susceptible to pain than others.

### Depression may impact pain through psychosocial pathways

Depression is highly prevalent among cancer survivors, with women being affected at twice the rate of men, even after a cancer diagnosis [5, 6]. Studies also find that rural cancer survivors report poorer mental health and more unmet mental health needs than nonrural survivors [15, 16]. The Stress Generation Hypothesis of depression postulates that symptoms of depression lead individuals to have negative thoughts, emotions, and behaviors that contribute to negative experiences in their lives [17, 18]. In women cancer survivors, depression may worsen pain severity by decreasing one’s self-efficacy, or the belief that they can effectively manage their health and overcome challenges. This may hold particular relevance to women cancer survivors who are dealing with age-related medical conditions and conditions caused by cancer therapies [19], as studies have shown that individuals who possess negative attitudes about themselves tend to experience greater pain severity [20, 21]. Depression is also marked by poor coping skills, which may contribute to women feeling overwhelmed by their responsibilities in life. This can lead to an increase in negative emotional states that can further amplify pain sensitivity and reduce pain thresholds, given evidence that older adults with high negative affect report frequent and severe pain [22]. Feeling overwhelmed can also lead women cancer survivors to neglect doing things that are necessary to manage their pain, such as navigating their health systems to obtain appropriate care. Additionally, people struggling with symptoms of depression often have communication and attributional styles that make them susceptible to interpersonal conflict. Whereas social support has been shown to help modulate pain severity [23], some studies in chronic health populations have found that social conflict, particularly with close others, directly contributes to individuals’ pain [24, 25]. Frequent and intense conflict with others may also diminish the quality of care provided to women cancer survivors, given the importance of both formal and informal caregivers to their health outcomes.

### The current study

The goal of the current investigation is to understand the relationship between symptoms of depression and pain severity in a sample of survivors of women’s cancer (breast, cervical, ovarian, or endometrial/uterine). We hypothesized that a higher level of symptoms of depression would be associated with more severe pain in the last week. We also hypothesized that the relationship between symptoms of depression and pain severity would be mediated by affective, cognitive, and interpersonal factors. Specifically, we hypothesized that symptoms of depression would be positively associated with feeling overwhelmed by life’s responsibilities, having low self-efficacy for managing health, and experiencing more relational burden.

## Methods

### Overview and participants

This is a secondary analysis of data collected from a trial whose primary purpose was to identify sociodemographic disparities in treatment burden among women cancer survivors. A detailed description of the recruitment procedures, study survey design, and identification of target population can be found in a prior paper [26]. A telephone survey interview was completed among 183 adult women survivors (*M*_age_ = 64 years old, SD = 12 years) primarily of breast, cervical, ovarian, or endometrial/uterine cancer, stages 1–3, and if they were between 6 months and 3 years post-completion of active therapy (surgery, chemotherapy, or radiation). Potential participants were identified from institutional registries at two large regional cancer centers in the mid-Atlantic US The time required to administer the eligibility screener and survey by phone averaged approximately 1 h. Table 1 contains participants’ demographics and health-related variables. Approximately half (46%) of the women interviewed resided in a rural setting, based on rural–urban commuting area (RUCA) county characteristics. More descriptives of the study sample can be found in the previous paper [26]. All participants provided their oral informed consent before participating in this study. This study was conducted in accordance with the Declaration of Helsinki and received ethical approval from the University of Virginia Institutional Review Board for Health Sciences Research (UVA IRB-HSR#19516).

**Table 1:** Patients’ (*N* = 183) demographics and health-related variables

*Demographic and health-related variables*	*n* (%) or *M* (SD)
Race	
White	167 (91%)
Other	6 (9%)
Latino(a)/Hispanic	5 (3%)
Household income	
Less than $35,000	54 (30%)
$35,000 to $75,000	44 (24%)
More than $75,000	65 (35%)
Education	
Less than high school	15 (8%)
Completed high school	32 (17%)
Some college	23 (13%)
Completed college (2 or 4 year degree)	58 (32%)
Beyond college	55 (30%)
Marital Status	
Married/living as married	125 (68%)
Single or divorced	58 (32%)
Employment status	
Working full-time	51 (28%)
Working part-time/not working	120 (65%)
Permanently disabled	12 (7%)
Cancer type	
Breast	106 (58%)
Cervical	9 (5%)
Ovarian	12 (7%)
Endometrial or uterine	51 (28%)
Other	5 (3%)
Number of pre-existing conditions	
0	46 (25%)
1	40 (22%)
2	31 (17%)
3	66 (36%)

Cumulative percentages for variables that do not equal 100% are due to patients choosing not to respond

### Measures

#### Symptoms of depression

Symptoms of depression were assessed using the 10-item version of the Center for Epidemiologic Studies Depression Scale (CES-D [27]). The CES-D-10 is a well-validated and accepted measure of symptoms of depression in cancer populations [28, 29]. Participants respond (0 = rarely or none of the time to 3 = all of the time) to 10 items that assess symptoms related to depression (e.g., I felt lonely and I felt depressed), based on how they have felt over the past week. In this study, CES-D scores ranged from 0 to 29 (*M* = 6.10, SD = 7.01) and internal consistency reliability was excellent (Cronbach’s *α* = 0.90). A cutoff score of 10 or greater, from a possible score range of 0 to 30, has been used to indicate a clinically significant level of depression in older adults [30]. Of the total sample, 45 survivors (25%) had a CES-D-10 score above the clinical cutoff.

#### Pre-existing health conditions

Cancer survivors were asked “before you were diagnosed with cancer, were you ever told by a healthcare provider that you had any of the following conditions?” with response options of diabetes, high blood pressure, high cholesterol, neuropathy, arthritis, depression, anxiety, or other preconditions. Approximately 75% of participants reported having at least one pre-existing condition. To reduce bias due to confounding, number of pre-existing conditions was entered as a covariate in the mediation models reported further below.

#### Pain

Pain was assessed using the single-item pain scale from the Patient Reported Outcomes Measurement Information System (PROMIS [31]). They were asked to rate their pain on average in the past 7 days on a scale ranging from 0 (no pain) to 10 (worst pain imaginable). Scores of the study sample ranged from 0 to 10 (*M* = 2.81, *SD* = 2.80).

#### Self-efficacy for managing health

We assessed self-efficacy in managing one’s chronic health condition with the shortened version of the Perceived Medical Condition Self-Management Scale (PMCSMS) [32], which is intended for use among patients with chronic disease. Survivors were read a series of statements “that might describe you and your healthcare.” For each one, they were asked (1 = strongly agree; 5 = strongly disagree) the degree to which they agreed with each of the following four statements: “It is difficult for me to find effective solutions for problems that occur with managing my medical condition,” “I am able to manage things related to my medical condition as well as most other people,” “No matter how hard I try, managing my medical condition doesn’t turn out the way I would like,” and “I’m generally able to accomplish my goals with respect to managing my medical condition.” Scores were summed such that higher scores indicated less confidence regarding their ability to manage their health. Scores ranged from 6 to 18 (*M* = 8.83, *SD* = 2.99). Internal consistency for this measure was acceptable (Cronbach’s *α* = 0.77).

#### Relational burden

We assessed relational burden using the 4-item interpersonal challenges scale of the Patient Experience with Treatment and Self-management (PETS) [33]. Survivors were instructed to think about their health and relationships with other people that they are close to (like family members, friends, or coworkers). They were then asked to rate how bothered they have been over the past 4 weeks by “feeling dependent on others due to your healthcare needs,” “others reminding you to do things for your health like take your medicines, watch what you eat, or schedule medical appointments,” “your healthcare needs creating tension in your relationships with others,” and “others not understanding your health situation.” All items were rated on a scale ranging from 1 (not at all bothered) to 5 (very bothered). Scores were summed such that higher scores indicated more relational burden. Scores ranged from 4 to 19 (*M* = 5.71, *SD* = 2.88). Internal consistency for this measure was acceptable (Cronbach’s *α* = 0.76).

#### Feeling overwhelmed by life’s responsibilities

We adapted six items from the Diabetes Distress Scale [34] to assess the extent to which cancer survivors felt overwhelmed by their life’s responsibilities which includes their health condition. Survivors were asked to “think about how much each of the following may have distressed or bothered you over the past 4 weeks” with items consisting of “feeling overwhelmed,” “not getting enough help from healthcare team,” “feeling like you’re failing to do what you should be doing,” “lack of support from friends and family,” “feeling you can’t do things you like to do,” and “feeling like your health problems are taking up too much of your energy every day.” To be consistent with the five-point response option systems of the PMCSMS [32] and PETS [33, 35], response options in this scale ranged from 1 (not at all bothered) to 5 (very bothered). Scores were summed such that higher scores indicated greater burden from responsibilities. Scores ranged from 6 to 30 (*M* = 10.68, *SD* = 5.57). Internal consistency for this measure was good (Cronbach’s *α* = 0.88).

### Plan for analysis

Zero-order correlations were computed to examine associations between symptoms of depression, pain, and mediational variables. For mediational analyses, an SPSS macro was used (PROCESS [36]) that employs a bootstrapping procedure to produce an estimate of effects and 95% confidence intervals based on 5000 resamples, with continuous scores of depression symptoms entered as the independent variable and pain severity entered as the dependent variable.^1^ Bootstrapping, a non-parametric method based on resampling with replacement, is commonly used when testing indirect effects such as in mediation analyses [37, 38]. To examine the role of individual variables in mediating the link between symptoms of depression and pain severity, mediating variables (i.e., feeling burdened from responsibilities, self-efficacy in managing health, and relational burden) were first tested separately in independent models. Then, to examine which of the variables would most strongly mediate the link between symptoms of depression and pain severity, all variables were entered simultaneously as mediators in the same model. To reduce potential bias due to confounding, the following variables were entered as covariates in each mediation model: number of pre-existing conditions, chemotherapy, age at diagnosis, race, education, marital status, employment, income, and work status.

Despite the frequent use of mediation models in the health sciences, there is no consensus way to estimate the sample size required to perform mediation analyses. A seminal paper by Fritz and MacKinnon [39] provides a guide for researchers who are interested in testing mediation models. In general, testing mediation models through bootstrapping (as done by PROCESS) requires a smaller sample size than the traditional causal steps model by Baron and Kenny [40]. Our sample size exceeds the minimum necessary to achieve 80% power assuming a small-to-moderate effect (> 0.26) of symptoms of depression on pain severity, a moderate effect of symptoms of depression on each of the mediators (α), and a moderate effect of the mediators on pain severity.

## Results

As seen in Table 2, there were significant positive associations between all variables. There was a significant positive association between symptoms of depression and pain severity, feeling more overwhelmed by life’s responsibilities, lower self-efficacy for self-management of health issues, and more relational burden.

**Table 2:** Zero-order correlations (and 95% confidence intervals)

	Pain severity	Low self-efficacy	Overwhelmed by life’s responsibilities	Relational burden
Symptoms of depression	.53** (.42, .63)	.59** (.48, .67)	.79** (.73, .84)	.73** (.67, .80)
Pain severity		.46** (.34, .57)	.58** (.47, .67)	.46** (.34, .57)
Low self-efficacy			.60** (.50, .69)	.52** (.40, .62)
Overwhelmed by life’s responsibilities				.79** (.72, .84)

**p < .01

**Fig. 1:**
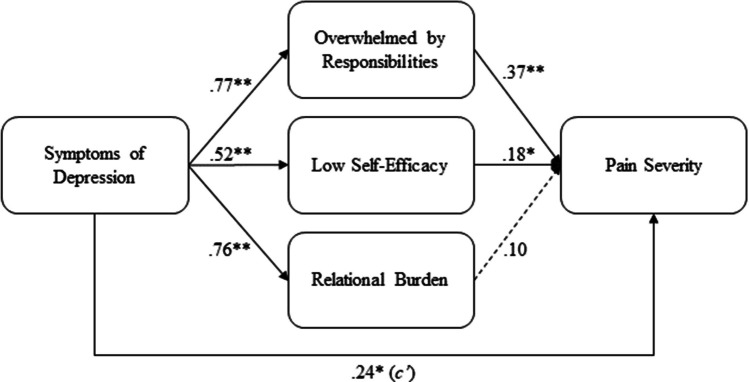
Standardized coefficients from individual mediation analyses. Note: the value of the *c’* path directly from symptoms of depression to pain severity is the average of the three *c’* paths from individual mediation analyses. The following variables were entered as covariates in each analysis: number of pre-existing conditions, chemotherapy, age at diagnosis, race, education, marital status, employment, income, and work status. **p < 0.01, * p < 0.05

As seen in Fig. 1, there was a significant direct effect (*c’* path) of symptoms of depression on pain severity from mediation analyses, such that higher levels of depression symptoms was directly associated with greater pain severity absent any mediators. Results from individual mediation analyses also revealed significant indirect effects of symptoms of depression on pain severity through feeling overwhelmed by life’s responsibilities (*B* = 0.28, *p* < 0.05, 95% CI = 0.09, 0.51) and lower self-efficacy for managing health (*B* = 0.09, *p* < 0.05, 95% CI =  − 0.01, 0.19), but not through relational burden (*B* = 0.07, 95% CI =  − 0.09, 0.22). Overall, women cancer survivors with higher levels of symptoms of depression felt more overwhelmed by life’s responsibilities and had lower self-efficacy for managing their health, which was associated with greater pain severity.

When all mediators were simultaneously entered into the same model, there was a significant indirect effect of symptoms of depression on pain severity through feeling burdened by responsibilities (*B* = 0.27, p < 0.05, 95% CI = 0.04, 0.51). Cancer survivors with higher levels of depression symptoms felt more overwhelmed by life’s responsibilities, which in turn was associated with greater pain severity, compared to those with lower levels of depression symptoms. Although cancer survivors with a history of depression also reported more relational burden and lower self-efficacy for managing their health, these variables were not significantly associated with pain severity.

## Discussion

Our understanding of how psychosocial vulnerabilities affect pain severity in women cancer survivors, and particularly those in rural settings, is limited. Our study reveals that a higher (vs. lower) level of symptoms of depression in women cancer survivors is associated with more severe pain. From individual mediation analyses, we also observed that low self-efficacy and feeling overwhelmed by life’s responsibilities mediate this relationship, which is consistent with vulnerability-stress models of depression in the general population [17, 41]. When all mediators were simultaneously tested in the same model, only feeling overwhelmed by life’s responsibilities emerged as significant, which indicates that this variable may be particularly relevant to understanding the relationship between symptoms of depression and pain severity. Overall, our study sheds light on potential pathways between depression and pain severity among cancer survivors. These findings may contribute to efforts to provide targeted support to women cancer survivors in rural areas at risk of developing severe pain through these pathways.

This study contributes to our understanding of pain severity in women cancer survivors by identifying potential mechanisms and pathways between symptoms of depression and pain. Despite literature on the relationship between depression and pain, there is a distinct lack of understanding of the mechanisms linking these variables [2]. Our findings suggest that symptoms of depression intensify pain severity in women cancer survivors through affective and cognitive pathways. This is consistent with research finding that individuals who are prone to depression tend to feel more overwhelmed and have lower self-efficacy than those who are more resilient [17, 18]. While our study suggests that a higher level of depression is linked to more severe pain due to lower self-efficacy for managing health and higher level of feeling overwhelmed, there may be more nuanced processes at play. For instance, low self-efficacy may lead some survivors to neglect their health due to the belief they are not deserving of care, which leads to greater pain severity due to deteriorating health, while for others it may lead to a belief that they are incapable of taking care of themselves despite a desire to remain healthy. Additionally, high negative affect can amplify pain sensitivity, which means that feeling more overwhelmed may lead to greater pain severity due to an increase in negative affect. Answering these questions will require studies that use intensive longitudinal designs to more frequently assess cancer survivors’ affect, beliefs, health behaviors, and their pain.

When all mediating variables were included in the same model, our findings suggest that feeling overwhelmed by life’s responsibilities is a crucial mediator in the link between symptoms of depression and pain severity. This is consistent with previous studies in non-cancer populations, which have found that, even at an early age, individuals vulnerable to depression may possess less effective coping skills to deal with stress and negative affect [42, 43]. Additionally, studies have consistently shown that women are more vulnerable to depression than men due to various biological and psychosocial factors, such as social support, societal norms, adverse life events, and coping styles [43]. As our study focused on women cancer survivors, feeling burdened by life’s responsibilities may be a unique factor in their pain severity. Despite major shifts in traditional gender roles, women still shoulder a larger share of household and childcare responsibilities than men in the US, even though they comprise almost half the workforce [44]. This may be particularly relevant to rural US households that tend to be more politically and culturally conservative [45]. Women cancer survivors who are experiencing symptoms of depression may be overwhelmed by managing their life responsibilities and cancer care, which could lead to neglecting necessary health behaviors to mitigate their pain. This process may be exacerbated in rural areas where access to mental healthcare is problematic and the stigma of receiving treatment for mental illness is prevalent [46, 47].

One avenue for future studies is to identify the impact of etiological factors in depression that may impact pain, such as genetic vulnerabilities and personality traits. Specifically, research has found that neuroticism may be a significant factor in the development and maintenance of depression [48, 49], which may contribute to poorer health outcomes in cancer survivors. Thus, even if a cancer survivor does not exhibit current signs and symptoms of depression, a high level of trait neuroticism may make them uniquely vulnerable to poorer health outcomes due to how they tend to think, feel, and behave [48, 49]. Understanding these vulnerability markers can help clinicians identify and target high-risk individuals for interventions that address pain, even if they are not displaying clinically significant symptoms of depression. By broadening our understanding of the underlying mechanisms of pain in cancer survivors, we can potentially address pain management and quality of life for those who are at greatest risk, thereby shifting focus from secondary to primary prevention strategies.

### Clinical implications

Additional research that illuminates the causal pathways between symptoms of depression and pain severity could lead to targeted interventions to address pain in cancer survivors before it worsens. For instance, psychological interventions, which have modest effects on pain severity in adults [50], lack the precision to guide when, where, how much, and under what circumstances the intervention should be optimally delivered for an immediate effect. Adaptive interventions that are personalized to the needs of individuals may produce stronger effects while reducing user burden [51, 52]. Due to the added complexity of these interventions versus the standard “one-size-fits-all” interventional approach, this is most easily accomplished in digital behavioral medicine due to the added flexibility and accessibility that is afforded through technology. Adaptive digital interventions may be especially relevant to rural cancer survivors given the lack of mental healthcare in those areas. An example of an adaptive digital interventional framework is one that continuously monitors individual survivors’ depression levels and pushes a brief behavioral intervention when depression exceeds a clinical threshold. Targeting depression early and often in women cancer survivors may attenuate key mediational factors and pain severity downstream. This kind of interventional framework would be possible through future research that defines the decision rules, decision points, tailoring variables, and proximal and distal outcomes of interest in this population [51].

### Study limitations

The limitations of the current study should be considered when interpreting its findings. The use of cross-sectional data limits the ability to draw conclusions about causality or directionality in the relationships between variables. Findings from this study are also based on a generally older sample of women who are more likely than younger women to be post-child rearing, post-menopausal, and either retired/not working or working on a part-time basis. Replication of these findings with longitudinal data and broader inclusion of younger women is necessary to establish causal relationships between depression, burden, and pain severity. These efforts may also include testing whether pain severity influence depression through mediators. Additionally, the current sample was limited to female, primarily White cancer survivors who received their cancer treatment at an NCI-designated Comprehensive Cancer Center with a large rural catchment area. Findings from this sample may therefore have limited generalizability to male, ethnic minority cancer survivors, or those who live in more urban areas. Finally, all measures in this investigation were based on cancer survivors’ self-report, which may inflate common method variance. Future studies should include more frequent and objective assessments of burden, interpersonal functioning, and pain, such as observer reports and passively collected data from survivors’ smartphone sensors. For example, studies have demonstrated the feasibility of using Bluetooth sensors to approximate social interaction [53], smartphone audio and camera inputs to detect pain [54], and multimodal physiological sensors from wearables to approximate emotional states [55].

To summarize, the current research elucidates potential pathways between symptoms of depression and pain severity in women cancer survivors. Our findings contribute to a larger body of literature and add to our understanding of the mechanisms underlying pain severity in cancer. With support from additional studies, these findings may have implications for how to identify and address pain in clinical practice. Further research should continue to explore the pathways linking variables related to psychological distress to pain severity in women cancer survivors.

## Data Availability

Data are available upon reasonable request.

## Footnotes

1 Note, the pattern of results for the mediation models is identical to those reported below when using depression status (0,1) as a dichotomous predictor, based on clinical cutoffs of the CES-D-10.

## Declarations

### Disclaimer

The findings in this manuscript represent the views of the authors and do not represent the official position of the NCI or NIH, USA.
